# Predicting diagnostic biomarkers associated with immune infiltration in Crohn's disease based on machine learning and bioinformatics

**DOI:** 10.1186/s40001-023-01200-9

**Published:** 2023-07-26

**Authors:** Wenhui Bao, Lin Wang, Xiaoxiao Liu, Ming Li

**Affiliations:** 1 Graduate School, Tianjin University of Traditional Chinese Medicine, Tianjin, China; 2 Spleen and Gastroenterology, Tianjin Academy of Traditional Chinese Medicine Affiliated Hospital, No.354 Beima Road, Hongqiao District, Tianjin, China; 3 Nephrology Department, First Teaching Hospital of Tianjin University of Traditional Chinese Medicine, Tianjin, China; 4 Department of Comprehensive Rehabilitation, First Teaching Hospital of Tianjin University of Traditional Chinese Medicine, Tianjin, China

**Keywords:** Machine learning, Immune infiltration, Biomarkers, Crohn's disease, GEO

## Abstract

**Objective:**

The objective of this study is to investigate potential biomarkers of Crohn's disease (CD) and the pathological importance of infiltration of associated immune cells in disease development using machine learning.

**Methods:**

Three publicly accessible CD gene expression profiles were obtained from the GEO database. Inflammatory tissue samples were selected and differentiated between colonic and ileal tissues. To determine the differentially expressed genes (DEGs) between CD and healthy controls, the larger sample size was merged as a training unit. The function of DEGs was comprehended through disease enrichment (DO) and gene set enrichment analysis (GSEA) on DEGs. Promising biomarkers were identified using the support vector machine-recursive feature elimination and lasso regression models. To further clarify the efficacy of potential biomarkers as diagnostic genes, the area under the ROC curve was observed in the validation group. Additionally, using the CIBERSORT approach, immune cell fractions from CD patients were examined and linked with potential biomarkers.

**Results:**

Thirty-four DEGs were identified in colon tissue, of which 26 were up-regulated and 8 were down-regulated. In ileal tissues, 50 up-regulated and 50 down-regulated DEGs were observed. Disease enrichment of colon and ileal DEGs primarily focused on immunity, inflammatory bowel disease, and related pathways. CXCL1, S100A8, REG3A, and DEFA6 in colon tissue and LCN2 and NAT8 in ileum tissue demonstrated excellent diagnostic value and could be employed as CD gene biomarkers using machine learning methods in conjunction with external dataset validation. In comparison to controls, *antigen processing and presentation, chemokine signaling pathway, cytokine–cytokine receptor interactions, and natural killer cell-mediated cytotoxicity* were activated in colonic tissues. *Cytokine–cytokine receptor interactions, NOD-like receptor signaling pathways, and toll-like receptor signaling pathways* were activated in ileal tissues. NAT8 was found to be associated with CD8 T cells, while CXCL1, S100A8, REG3A, LCN2, and DEFA6 were associated with neutrophils, indicating that immune cell infiltration in CD is closely connected.

**Conclusion:**

CXCL1, S100A8, REG3A, and DEFA6 in colonic tissue and LCN2 and NAT8 in ileal tissue can be employed as CD biomarkers. Additionally, immune cell infiltration is crucial for CD development.

## Introduction

**Fig. 1:**
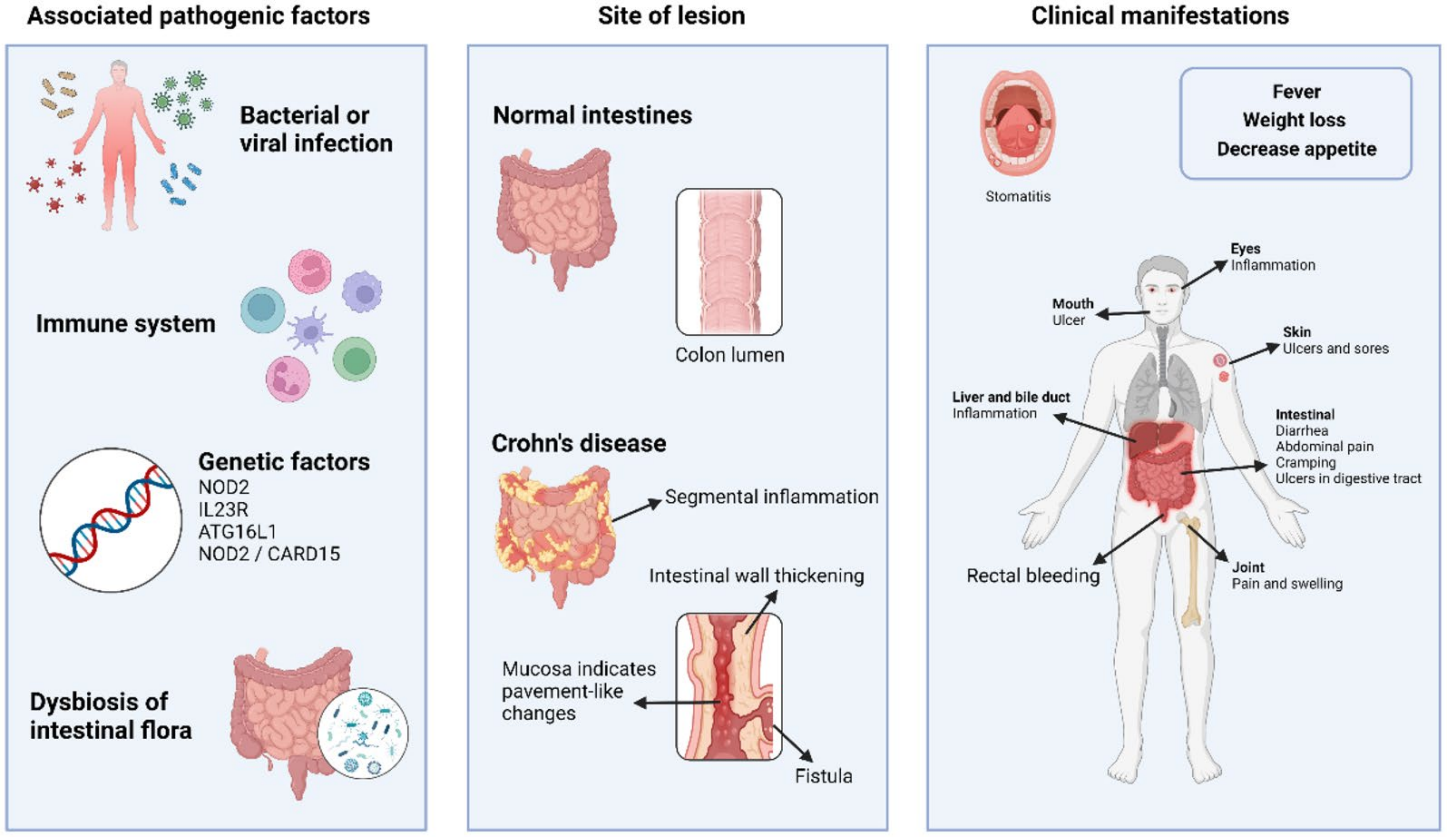
Pathogenesis, lesion components and clinical manifestations of CD

Crohn's disease (CD), a chronic, recurrent inflammatory bowel disease, is characterized by abdominal pain, diarrhea, blood in the stool, and weight loss. The disease alternates between periods of recurrence and remission and can be disabling. Its transmural inflammation most commonly affects the terminal ileum and adjacent colon [1], but it can involve any part of the gastrointestinal tract, from the oral cavity to the perianal area [2]. Some patients may experience extra-intestinal manifestations, such as iridocyclitis and erythema nodosum [3].The incidence of CD ranges from 3 to 20 cases per 100,000 people [4] and is increasing annually in most parts of the world, causing significant suffering and economic burden for patients. Currently, there are challenges in the early diagnosis and prevention of CD [5]. Diagnosis can only be made through a combination of patient history, imaging, and relevant ancillary tests [6].The pathogenesis of CD remains unclear but is closely related to the immune system, including factors such as infection, humoral and cellular immunity, genetic predisposition, and dysbiosis of the intestinal flora [7]. The genetic component of CD appears to be stronger in IBD than in UC, and CD is closely related to NOD2, IL23R and ATG16L1 genes [8, 9] (Fig. 1). The NOD2/CARD15 gene is not only associated with ileal damage, fibrous stenosis, and a family history of CD, but also increases the risk of developing the disease [10]. Concurrently, research related to immunomodulation in CD is increasing, and studies suggest that CD is a progressive disease with periods of immune changes mediated [11]. CD is an immune-mediated enteropathy characterized by abnormal activation and infiltration of multiple immune cells, leading to the pathogenesis of inflammation and tissue damage in the intestine [1, 2]. Neutrophils play a crucial role in the initial stages of intestinal inflammation, exhibiting a substantial increase in both their quantity and activity. They release various inflammatory mediators that can impair the function of the epithelial barrier, thereby triggering an inflammatory response [12, 13]. The presence of neutrophil infiltration within the intestinal mucosa suggests the involvement of adaptive immunity [14]. In the pathogenesis of CD, macrophages and dendritic cells play crucial roles as important members of the immune cell population [15]. They are involved in antigen presentation and immune regulation [6, 7]. CD4 + T cells are a specific subclass of T lymphocytes. Upon activation, CD4 + T cells can differentiate into two distinct types: effector T cells and regulatory T cells [16]. An imbalanced ratio of these T cell subtypes in CD contributes to the development and worsening of inflammatory responses [17]. In the later stages of CD pathogenesis, there is an aberrant activation and proliferation of effector T cells, resulting from abnormal immune cell activity. These activated T cells mount an attack on the intestinal wall, leading to tissue damage and inflammation [5, 17]. Meanwhile, there is a decrease in the number and function of regulatory T cells (Tregs), which are primarily responsible for suppressing excessive immune responses and maintaining immune homeostasis. This imbalance within the immune system consequently leads to inflammation and tissue damage in the intestinal wall [18] Additional immune cells associated with CD in the mucosa include natural killer cells (NK) and natural killer T cells (NKT) [19]. Studies have shown that the balance of NK cells expressing NKp44( +) and NKp46( +) markers is disrupted in the intestinal mucosa of CD patients [20]. Consequently, it becomes evident that the precise regulation of immune cells and the maintenance of immune homeostasis are crucial for both the prevention and treatment of CD.

Early diagnosis and stratification based on disease localization is essential for the management of CD. CD is recognized as a progressive condition characterized by a period of immune-mediated changes. At the time of diagnosis, intestinal damage and immune dysregulation have typically already occurred, and in most cases medications cannot reverse existing intestinal damage [21]. However, more favorable outcomes may be achievable if the disease is diagnosed early, before significant intestinal damage develops in the initial stages. Timely diagnosis and treatment of the disease can significantly impact its course, promoting healing of the mucosa and reducing damage caused by hospitalization or surgical intervention [9, 22, 23]. Current treatment of CD not distinguish between small bowel CD and ileal CD and the location of disease onset influences the prognosis of disease progression [24]. For example, the microbiota is more disrupted in ileal than in colonic CD; the probability of fibrotic stenosis is higher in ileal CD than in colonic CD, and the risk of surgery is higher than in colonic CD [25]. Relevant data also show that there is a correlation between the efficacy of biologic agents and the site of CD [26]. While different locations and disease progressions usually necessitate varying treatments, the pathophysiological mechanisms underlying the differentiation between colonic CD and ileal CD remain unresolved.

Based on the above-mentioned CD pathogenesis, diagnosis and treatment status, this study screens for ileal and colonic related CD diagnostic biomarkers and searches for potential therapeutic targets based on immune infiltration, respectively. The attempt is to stratify patients according to CD disease localization and to better individualize the treatment of patients. In this study, we obtained the gene expression matrix of CD from the GEO database using a bioinformatics approach. The dataset was divided into two groups based on the site of CD's lesion: colonic and ileal. To identify CD-related biomarkers, we employed two machine learning algorithms, namely LASSO and SVM-RFE. Subsequently, candidate genes that showed a close association with immune infiltration were further validated using an independent validation cohort. CIBERSORT was used to quantify the ratio of immune cells in CD and normal tissue samples based on gene expression profiles, and to analyze and screen the relationship between infiltrating immune cells and relevant biological markers, providing a reference for the prevention and treatment of CD.

## Materials and methods

### Acquiring microarray data

Screening was performed in the GEO database using "Crohn's disease" as the search phrase, limiting the entry type to "series", study type to "expression profiling by array", tissue source organism to "Homo sapiens", and sample size to > 50. All genetic expression data related to CD were retrieved up to September 1, 2022. Inflammatory lesion tissues from Crohn's patients were selected and differentiated into colon and ileum. A total of three eligible gene expression datasets were screened (GSE75214, GSE20881, GSE179285). GSE75214 contains 8 CD and 11 control samples from colon tissue, as well as 51 CD samples and 11 controls from ileum. GSE20881 comprises 34 CD and 67 control samples from colon tissue and 7 CD and 6 control samples from ileum tissue. GSE179285 includes 14 CD and 23 control samples from colon tissue and 33 CD and 8 control samples from ileum tissue.

### Data filtering and processing

The downloaded probe matrix was converted into a gene expression matrix according to the probe annotation file. When a gene was associated with more than one probe, the mean value of the probes was determined to reflect the ultimate expression level of the gene. In the colonic group, GSE20881 was combined with GSE179285 to form a training group, while GSE75214 served as a validation group. In the ileal group, GSE75214 was merged with GSE179285 as the training group, and GSE20881 was used as the verification group. Batch effects were addressed using the SVA package, and differences in the expression matrix between the control and experimental groups were analyzed using the limma package. To identify immune infiltration-related diagnostic gene expression profiles in CD patients, |log FC|> 2 and adjusted *P* value < 0.05 were the criteria used to discover the DEGs. The volcano plots were generated using ggplot.

### Analysis of functional enrichment

An enrichment analysis of disease ontology (DO) was conducted on the DEGs to investigate the diseases in which they were enriched. The analysis was carried out using the clusterProfiler, org.Hs.eg.db, DOSE, and enrichplot packages, with the "c2.cp.kegg.v7.4.symbols.gmt" database as a reference. P values less than 0.05 were used to determine whether a pathway was significantly enriched.

### Machine learning for identifying potential biomarkers

Machine learning is a novel tool for algorithmic analysis. In this study, the least absolute shrinkage and selection operator (LASSO) and support vector machine-recursive feature elimination (SVM-RFE) were combined to identify CD diagnostic biomarkers. In the LASSO regression algorithm, we used the "glmnet" package in R for identification and cross-validation. The SVM-RFE algorithm was employed to screen the gene set most associated with CD. By taking the intersection of the significant genes identified by both techniques, diagnostic biomarkers for the disease were discovered.

### Diagnostic value validation for potential biomarkers

The diagnostic biomarkers identified by machine learning were validated for accuracy in the validation group, and boxplots and receiver operating characteristic (ROC) curves were plotted. The greater the area under the ROC curve (AUC), the higher the accuracy.

### Analysis of immunity cell infiltration and correlation

CIBERSORT is a linear support vector regression (SVR)-based machine learning method with advantages in identifying human immune cell phenotypes [27]. CIBERSORT was used to obtain the relative amounts of immune cells in each sample, determining the relative proportions of immune cells in CD. Correlations between immune cells were analyzed and visualized using the "corrplot" package. The "vioplot" R package was applied to create violin plots to display the differences in immune cell infiltration between the two groups. Spearman correlation coefficients were used for the investigation of correlations between diagnostic gene biomarkers and immune cells, and "ggplot2" was employed to visualize the results.

### Statistical analysis

The Mann–Whitney *U* test was employed for continuous variables involving two groups with a non-normal distribution. For continuous variables comparing three groups, ANOVA was used. The association between immune cell percentage and gene expression was examined using Pearson analysis. The effectiveness of the study's identified diagnostic indices was evaluated using ROC curve analysis. R software and SPSS software were utilized for all statistical analyses.

## Results

### Identify DEGs results in CD

**Fig. 2:**
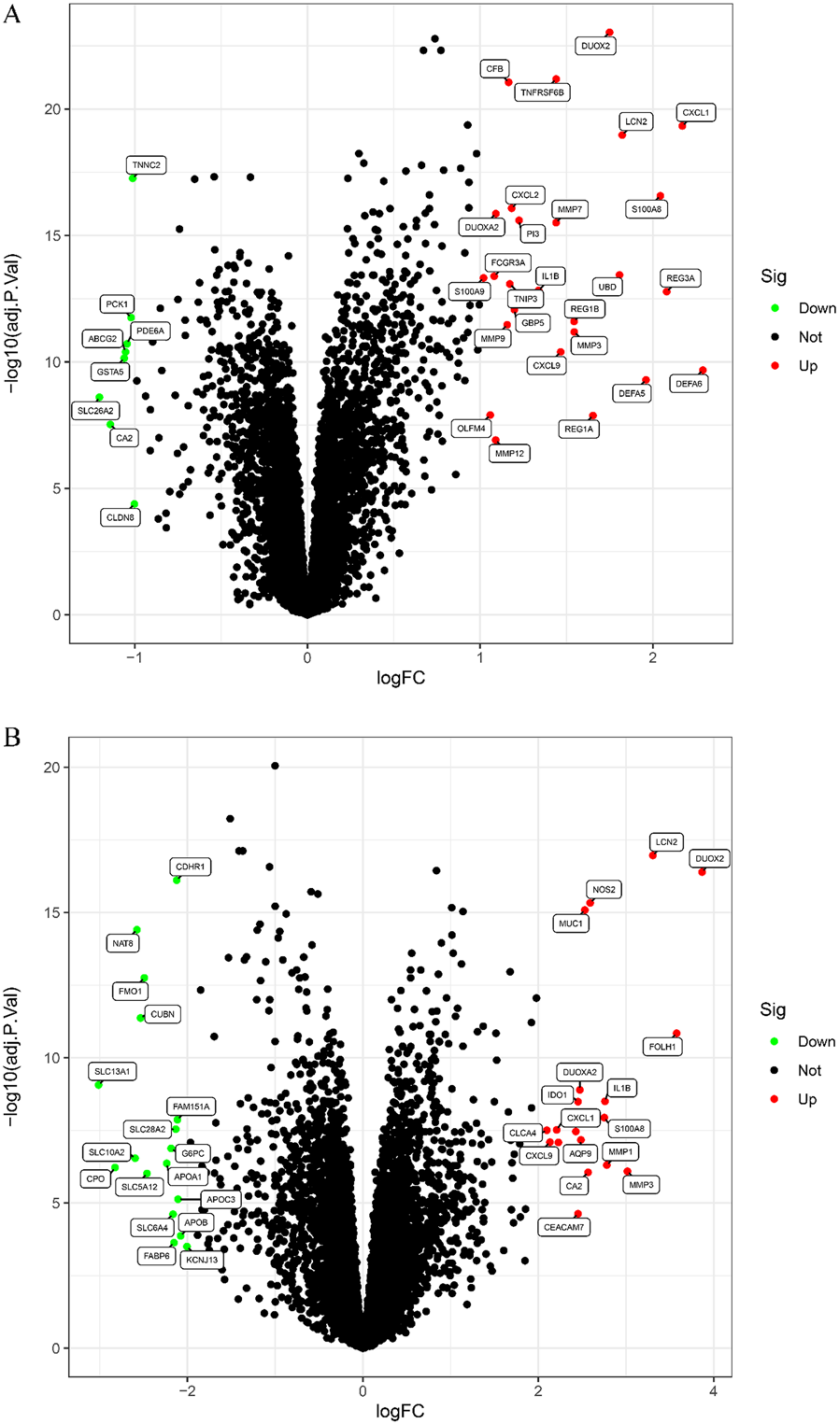
Volcano map comparing CD samples to healthy samples. **A** Colon tissue; **B** ileal tissue. The vertical axis denotes the importance of the distinction, whereas the transverse axis shows the variance multiplier in gene expression between CD and the control sample

Significantly differentially expressed genes (DEGs) were screened out in the colon group and ileum group, respectively. The screening conditions were adjusted *p*-value < 0.05 and |log fold change (FC)|> 2. DEGs volcano plots were created accordingly. In the colon group, 34 significant DEGs were identified, with 26 considerably up-regulated genes including DUOX2, TNFRSF6B, CFB, CXCL1, LCN2, S100A8, CXCL2, DUOXA2, MMP7, UBD, FCGR3A, S100A9, IL1B, GBP5, REG1B, MMP9, CXCL9, DEFA6, DEFA5, OLFM4, MMP12, PI3, TNIP3, REG1A, MMP3, and REG3A. Eight genes were significantly down-regulated: TNNC2, PCK1, ABCG2, PDE6A, GSTA5, SLC26A2, CA2, and CLDN8 (Fig. 2A). In the ileocecal group, 34 DEGs were screened, with 17 showing significantly up-regulated expression, including LCN2, DUOX2, NOS2, MUC1, FOLH1, DUOXA2, IL1B, IDO1, CXCL1, CLCA4, S100A8, CXCL9, AQP9, MMP1, CA2, MMP3, and CEACAM7. Meanwhile, 17 genes showed significantly down-regulated expression: CDHR1, NAT8, FMO1, CUBN, SLC13A, FAM151A, SLC28A2, SLC10A2, G6PC, CPO, SLC5A12, APOA1, APOC3, APOB, SLC6A4, FABP6, and KCNJ13 (Fig. 2B).

### Enrichment results of DEGs

#### DO enrichment analysis

**Fig. 3:**
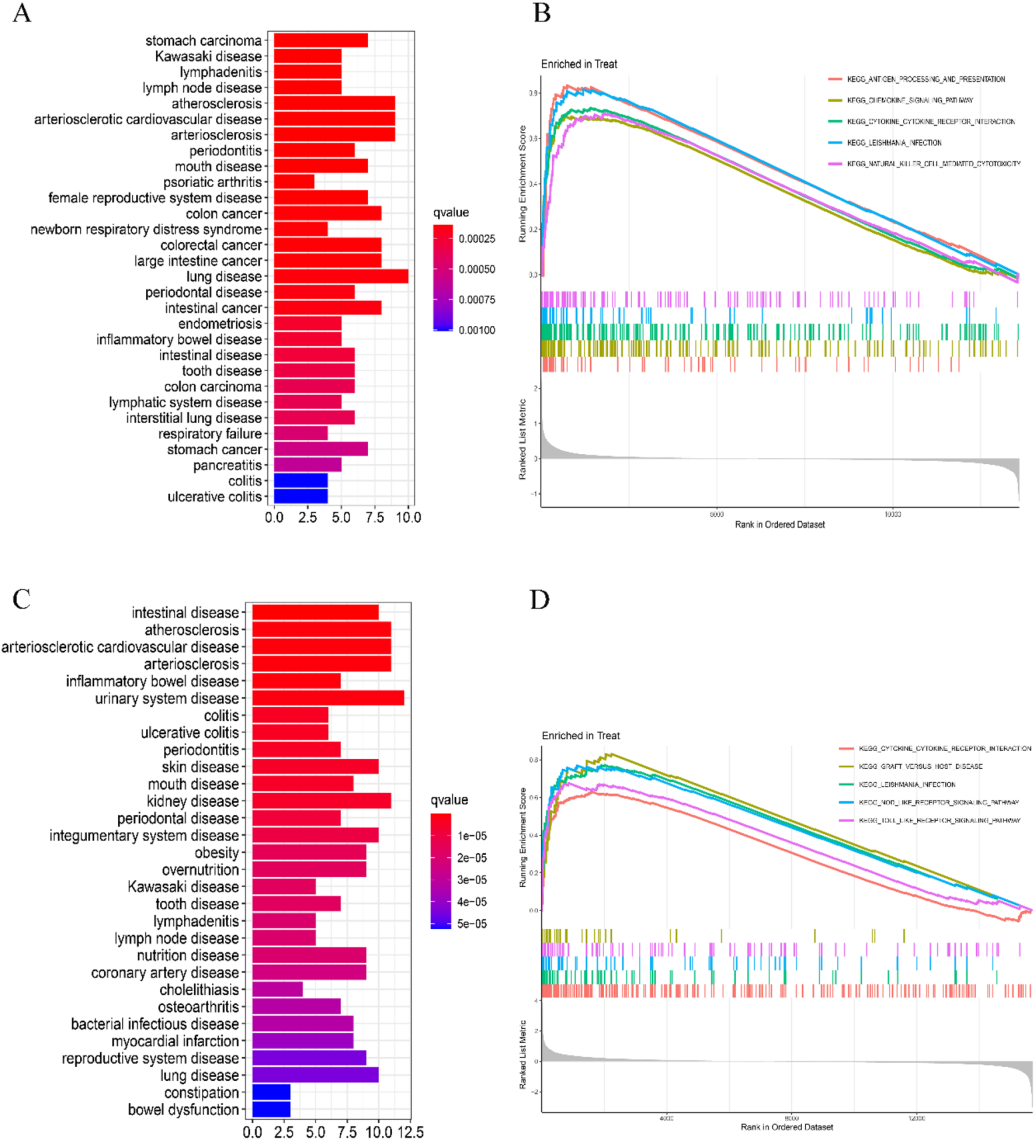
Potential biological processes for functional enrichment analysis of CD DEGs. **A**, **C** DO enrichment analysis of DEGs. **B**, **D** GSEA enrichment analysis of DEGs. **A**, **B** Colon tissue; **C**, **D** ileal tissue

In colon tissue, DEGs of CD were primarily enriched in diseases such as intestinal cancer, immune-related diseases, oral diseases, and lung diseases (Fig. 3A). In ileal tissue, DEGs of CD were primarily enriched in intestinal diseases, oral diseases, colonic diseases, and inflammatory bowel diseases (Fig. 3C). The *p*-values were all less than 0.01.

#### GSEA set enrichment analysis

In colon tissue, the top five pathways enriched for CD compared with healthy controls were *antigen processing and presentation, chemokine signaling pathway, cytokine–cytokine receptor interactions, leishmaniasis infection, and natural killer cell-mediated cytotoxicity* (Fig. 3B). In ileal tissue, the top five pathways enriched for CD genes compared to healthy controls were *cytokine–cytokine receptor interactions, graft-versus-host disease, leishmaniasis infection, NOD-like receptor signaling pathways, and Toll-like receptor signaling pathways*, in that order (Fig. 3D).

### Validation of biopotential biomarkers

**Fig. 4:**
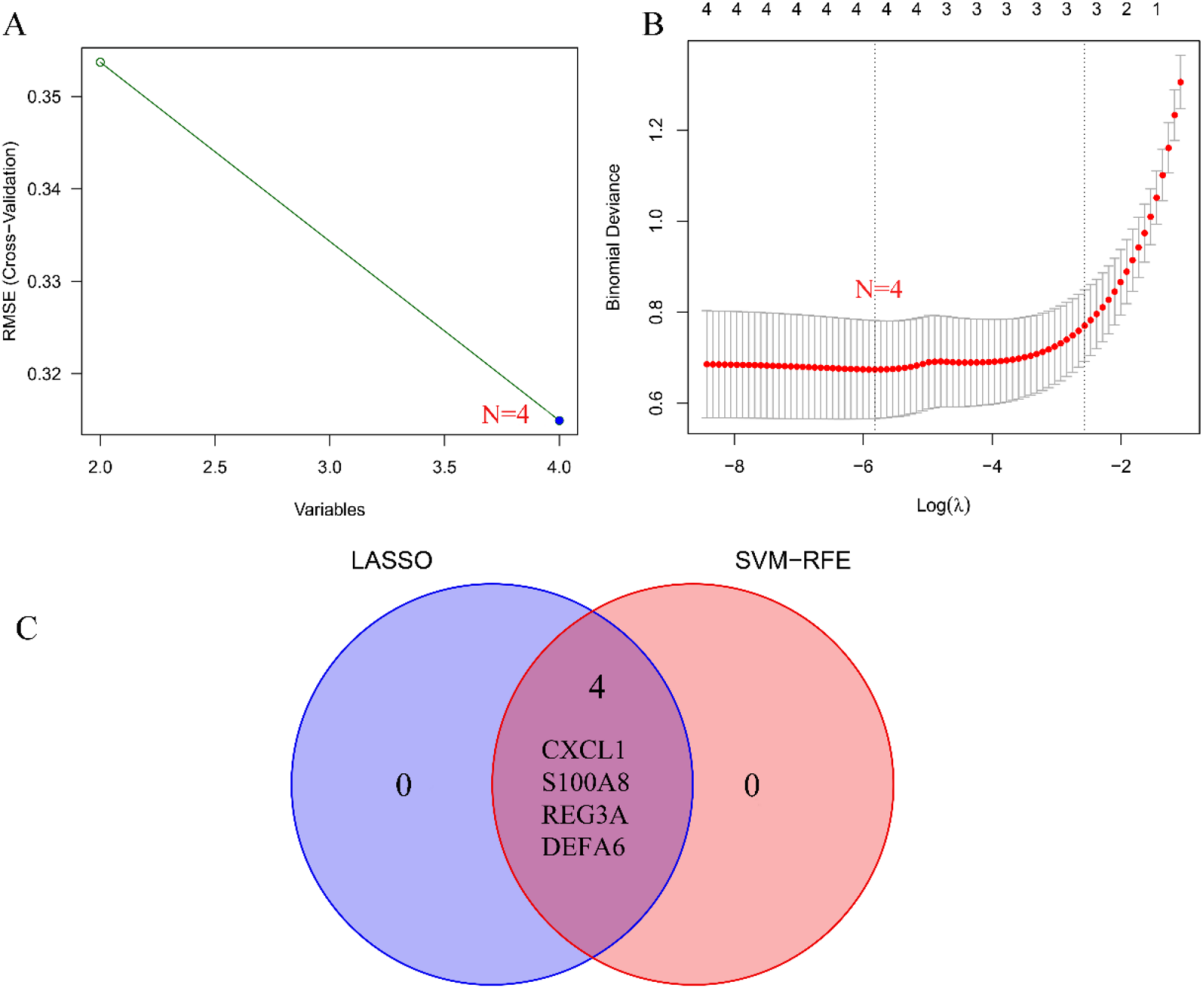
Identifying potential biomarkers for CD. **A** The SVM-RFE model. The horizontal axis denotes the number of featured genes, while the vertical axis represents the error rate in curve variation after cross-validation. In the graph, *N* = 4 in the figure indicates that there are 4 feature genes with the lowest error rate, which is close to zero. **B** The LASSO model. The horizontal axis displays the logarithmic punishment coefficient, log *λ*, while the vertical axis shows the error of the cross-validation. A lower value on the *Y*-axis indicates a better fitting result of the equation. The two dashed lines indicate two specific lambda (*λ*) values. The dashed line on the left represents *λ* min, which indicates the lambda value when the bias is minimal, signifying that the model fitting is the best at this lambda value. In this study, *λ* min on the left was chosen as the final criterion for selecting the equation. The dashed line on the right represents *λ*-se, which refers to one standard error to the right of the minimum *λ* value; **C** in colon tissue, LASSO and SVM-RFE share biomarkers

**Fig. 5:**
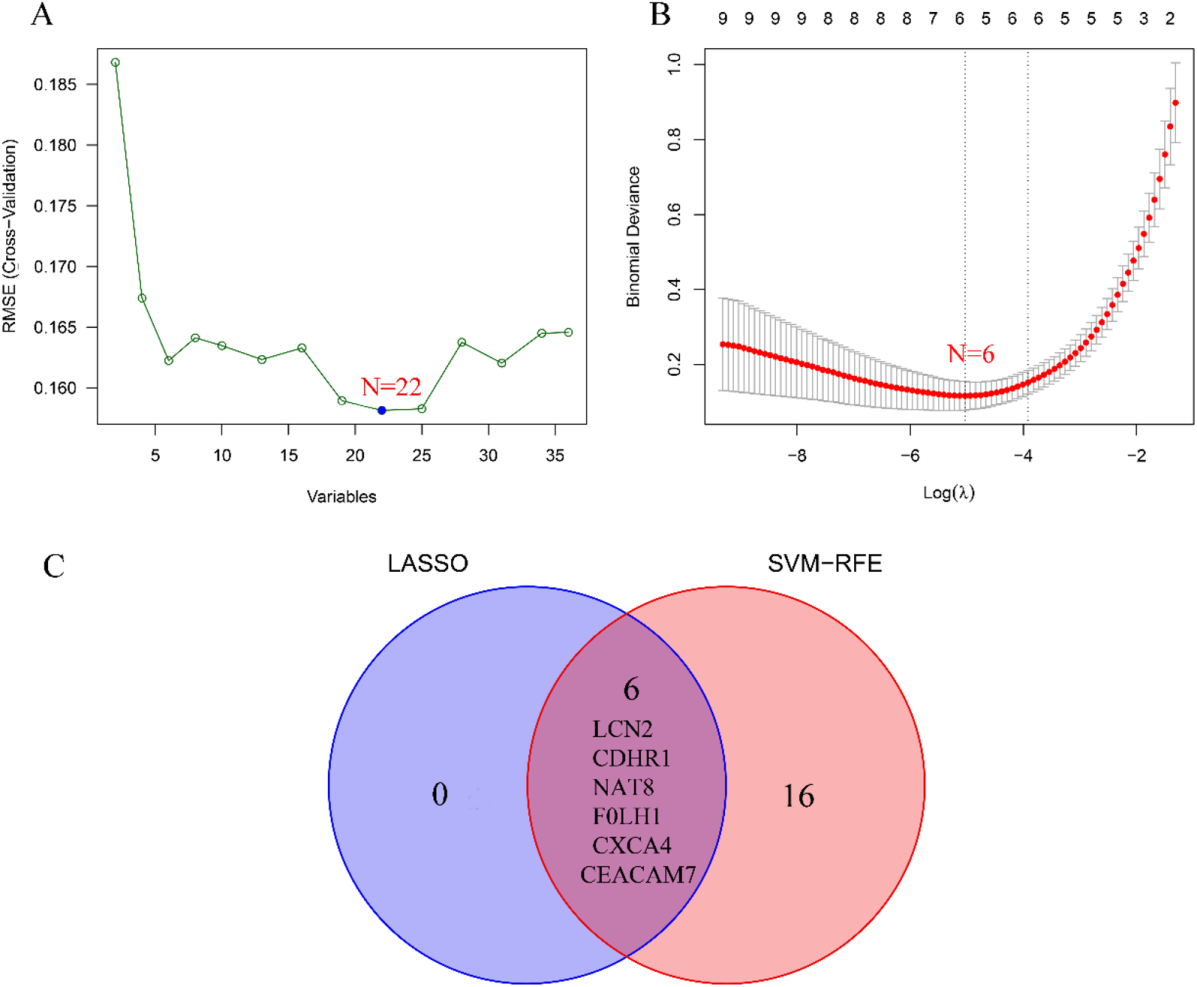
Identifying potential biomarkers for CD. **A** The SVM-RFE model; **B** the LASSO model; **C** in ileal tissue, LASSO and SVM-RFE share biomarkers

**Fig. 6:**
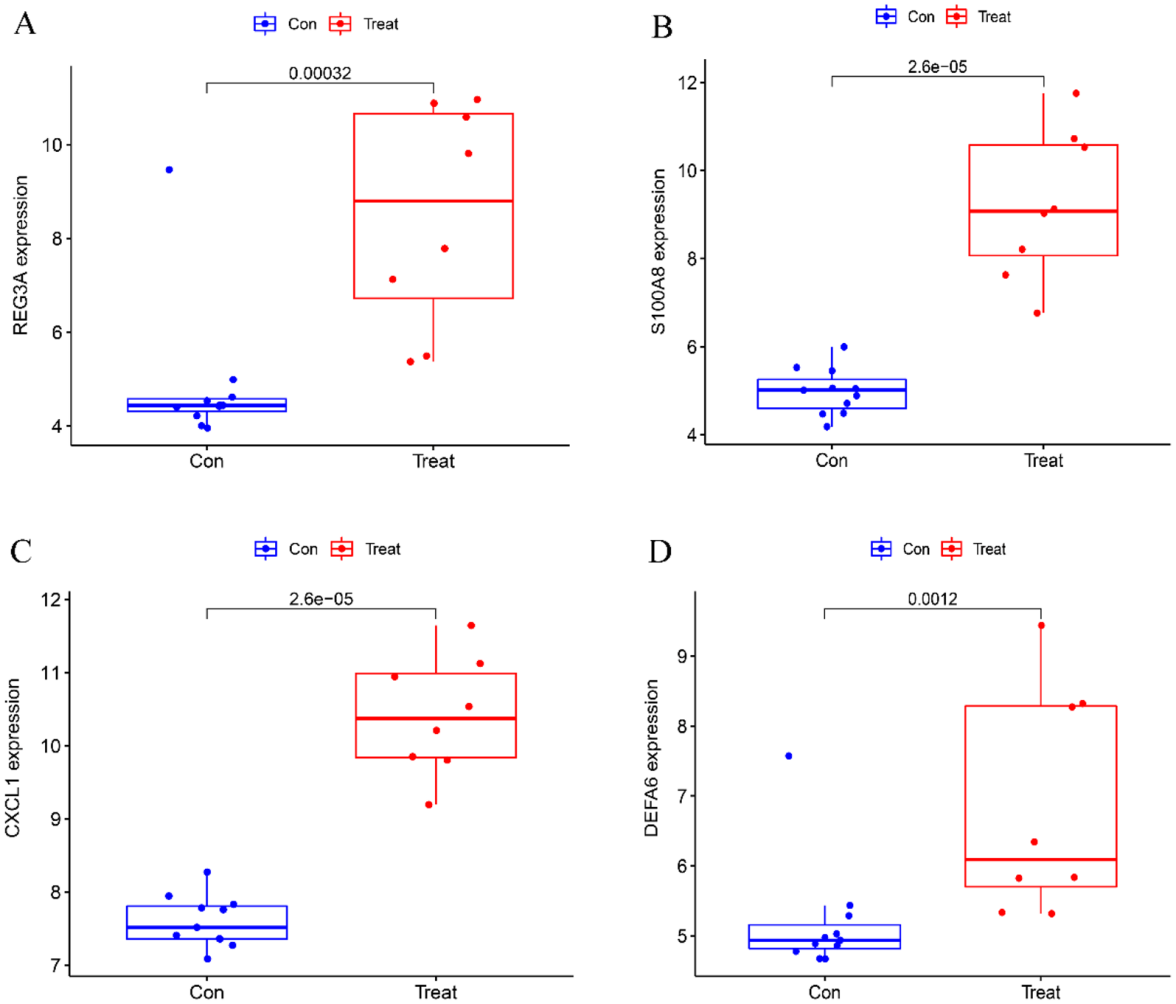
Validation of the expression of a diagnostic biomarker in the dataset for the validation group GSE75214. A (REG3A); B (S100A8); C (CXCL1); D (DEFA6)

**Fig. 7:**
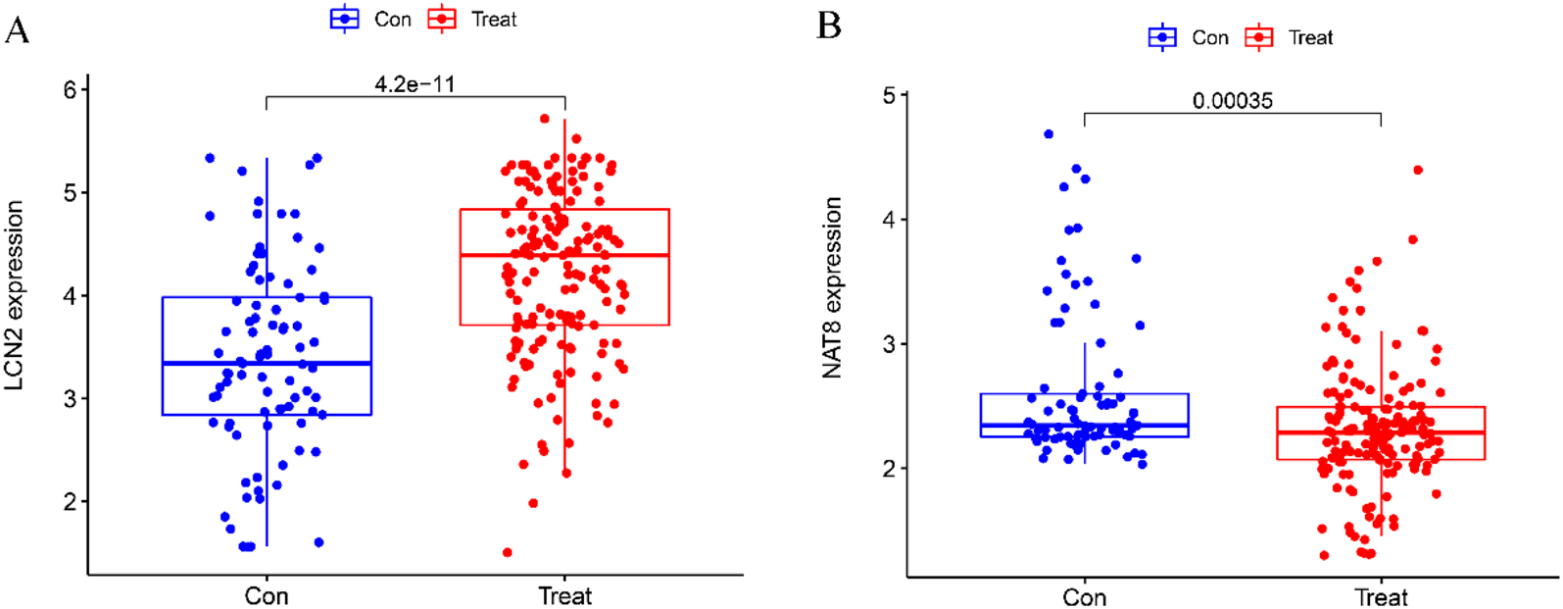
Validation of the expression of a diagnostic biomarker in the dataset for the validation group GSE20881. **A** (LCN2); **B** (NAT8). On a number axis, the box plot's upper and lower edges represent the upper quartile and lower quartile, respectively, enabling observation of the quartile distance to determine if normal value distribution is concentrated or dispersed. The median is represented by a thickened line in the middle of the box plot

To screen biomarkers, support vector machine (SVM-RFE) and LASSO regression, two machine learning techniques, were employed. In colon tissue, LASSO regression and SVM-RFE identified four biomarkers each (Fig. 4A, B). The intersecting genes CXCL1, S100A8, REG3A, and DEFA6 were determined to be diagnostic biomarkers (Fig. 4C). In ileal tissue, LASSO regression identified six biomarkers (Fig. 5A), while SVM-RFE identified 22 biomarkers (Fig. 5B). The intersecting genes of the two algorithms, LCN2, CDHR1, NAT8, FOLH1, CLCA4, and CEACAM7, were recognized as the six biomarkers (Fig. 5C). To verify the accuracy of the diagnostic biomarkers, further validation was performed separately in colon tissue and ileal tissue validation groups. In comparison to the control group, the colon group exhibited significantly higher expression levels of the four CD diagnostic biomarkers (*p* < 0.01) (Fig. 6A–D). The expression of the diagnostic biomarker LCN2 was markedly increased in the ileum group (*p* < 0.01) (Fig. 7A). Conversely, NAT8 expression was significantly reduced in the ileum (*p* < 0.01) (Fig. 7B).

### Diagnostic value of diagnostic biomarker

**Fig. 8:**
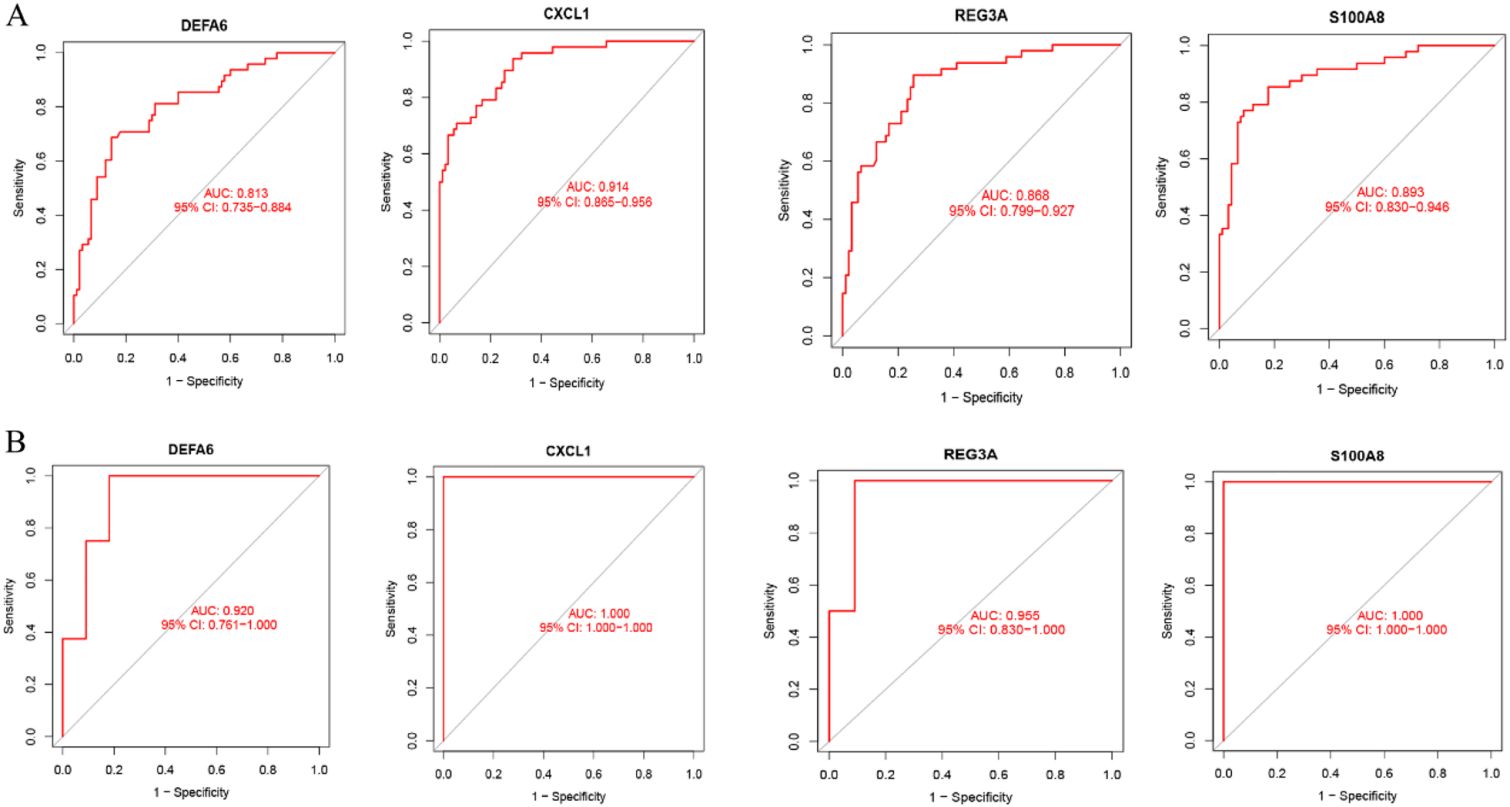
ROC curves of diagnostic validity of CD biomarkers. **A** The original data in the queue DEFA6, CXCL1, CXCL1, REG3A, S100A8 fitting variable after the ROC curve. **B** ROC curve of CXCL1,CXCL1, REG3A and S100A8 in GSE75214 database after fitting one variable

The AUCs of the four biomarkers screened in the training group in colon tissue were CXCL1 (0.914, 95% CI: 0.865–0.956), DEFA6 (0.813, 95% CI: 0.735–0.884), REG3A (0.868, 95% CI: 0.799–0.927), S100A8 (0.893, 95% CI: 0.830–0.946); see Fig. 8A. The AUCs of the screened diagnostic genes in the validation team were CXCL1 (1.000, 95% CI: 1.000–1.000), DEFA6 (0.920, 95% CI: 0.761–1.000), REG3A (0.955, 95% CI: 0.830–1.000), and S100A8 (1.000, 95% CI: 1.000–1.000) (Fig. 8B).

**Fig. 9:**
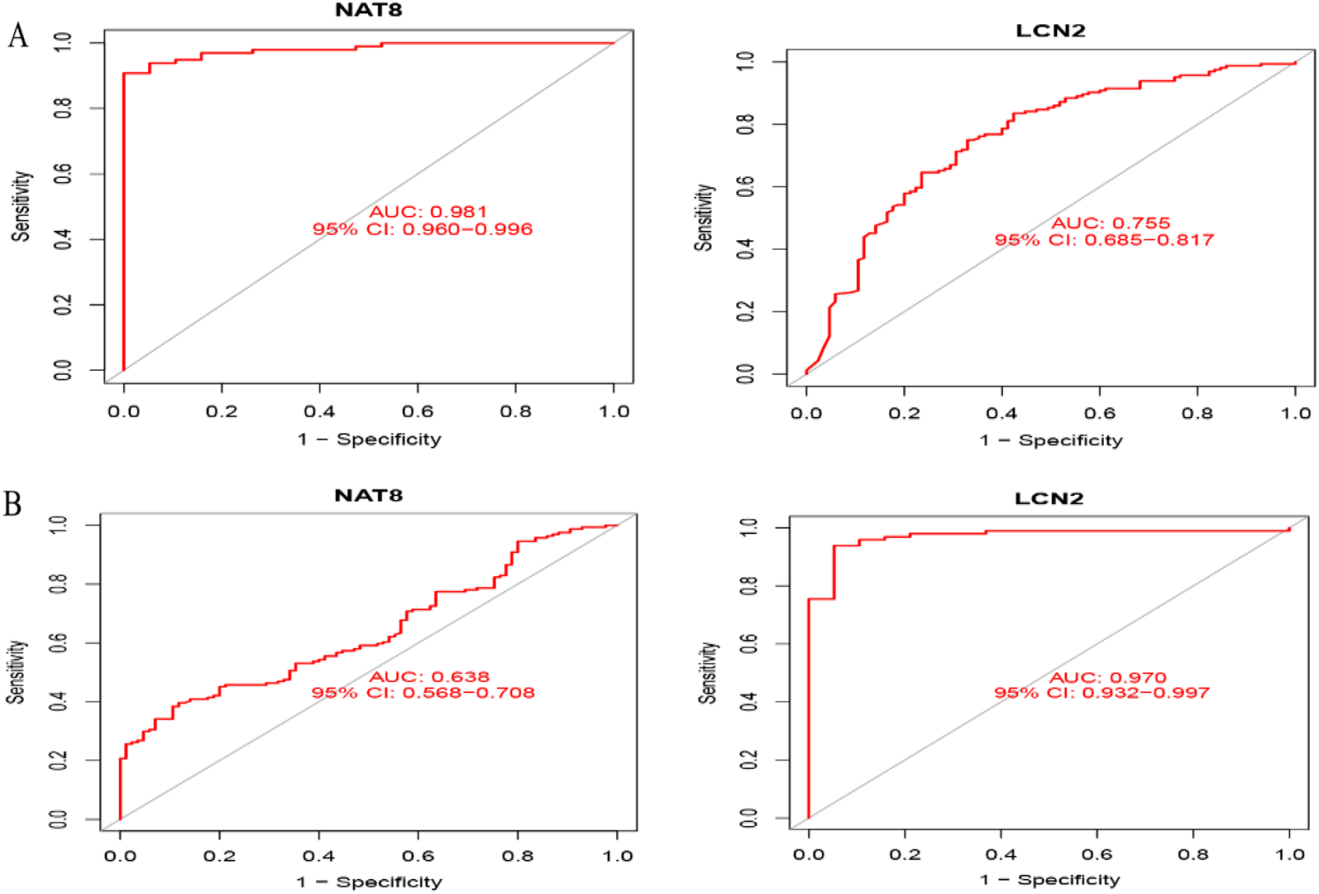
ROC curves for CD biodiagnostic marker diagnostic validity. **A** ROC curves after adjusting a variable to NAT8, LCN2 in the original data cohort. **B** ROC curves after setting NAT8, LCN2 to a variable in the GSE75214 database

The AUCs of the six diagnostic genes screened in ileal tissue were LCN2 (0.970, 95%, CI: 0.932 − 0.997), NAT8 (0.981, 95% CI: 0.960 − 0.996), CDHR1 (0.976, 95% CI: 0.948 − 0.997), CEACAM7 (0.939, 95% CI: 0.892 − 0.976), CLCA4 (0.904, 95% CI: 0.838 − 0.961), FOLH1 (0.923, 95% CI: 0.870 − 0.966). Among them, the ROC curves of LCN2, NAT8 are shown in Fig. 9A. The AUCs in the validation group for the diagnostic genes in the validation group were LCN2 (0.755, 95% CI: 0.685 − 0.817), NAT8 (0.638, 95% CI: 0.568 − 0.708) (Fig. 9B). The AUC of the diagnostic gene was basically greater than 0.7 in colon and ileal tissues, which had a high diagnostic value.

### Analysis of immune cell infiltration

**Fig. 10:**
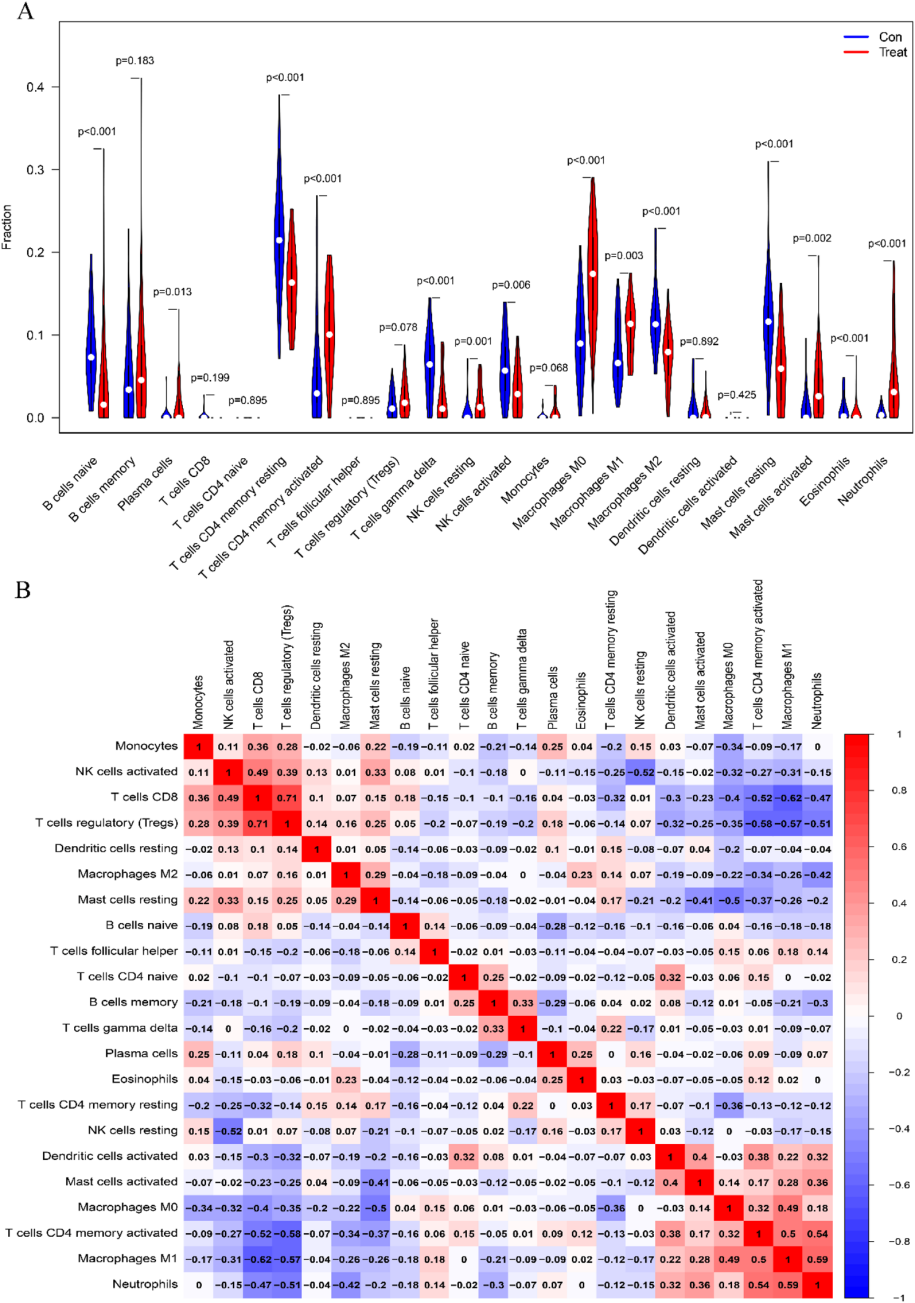

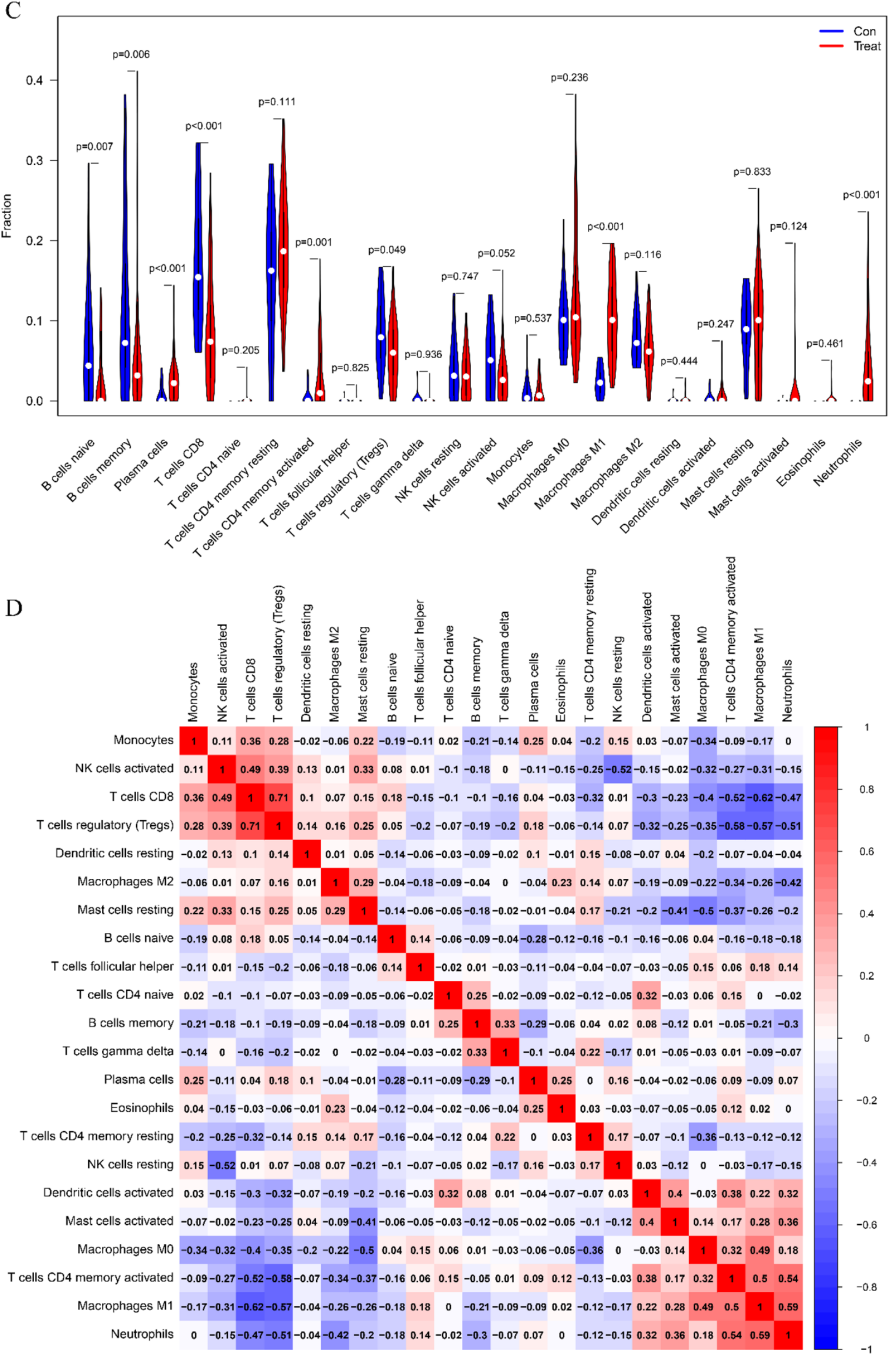
Immune cell infiltration distribution and visualization. **A**, **C** 22 immune cell subtypes in CD and normal tissues are compared. Red denotes the experimental CD group and blue the control group. **B**, **D** Heat map showing the relationships between 22 immune cell subtypes. Immune cell subtypes are displayed along both the horizontal and vertical axes, and the numbers inside correspond to the correlation coefficients of those immune cells. Positive and negative correlations are denoted by the colors red and blue, respectively. The strongest positive association between the two genes is shown by the darkest red cells, and the strongest negative correlation is represented by the darkest blue cells

*Neutrophils*, *B cells naive*, *eosinophils*, and *macrophages M0* were significantly higher in CD samples from the colonic group than in normal samples (*p* < 0.001), while *T cells CD4 memory resting*, *T cells CD4 memory activated*, *T cells gamma delta*, *macrophages M2*, and *mast cells resting* were significantly lower than in normal samples from the colonic group (*p* < 0.001) (Fig. 10A). *Neutrophils*, *macrophages M1*, *plasma cells*, *memory-activated T cells CD4*, and *T cells CD8* were all considerably higher in the ileal group compared to the normal group, but *T cells CD8* were significantly lower (*p* < 0.001) (Fig. 10C).

Furthermore investigated at was the linkage of 22 immune cells in all samples. T cells CD8 showed a strong positive link with *T cells regulatory* (Tregs) in CD colon tissue and normal samples (*r* = 0.71), while *T cells CD4 memory activated* showed a strong negative association with *T cells regulatory* (*r* = − 0.58) (Fig. 10B). *T cells CD8* displayed a substantial positive association with *T cells regulatory (Tregs)* in samples from CD ileus and healthy individuals (*r* = 0.71), while *T cells CD8* displayed a significant negative correlation with *Macrophages M1* (*r* = − 0.62) (Fig. 10D).

### Biomarker and infiltrating immune cell correlation analysis

**Fig. 11:**
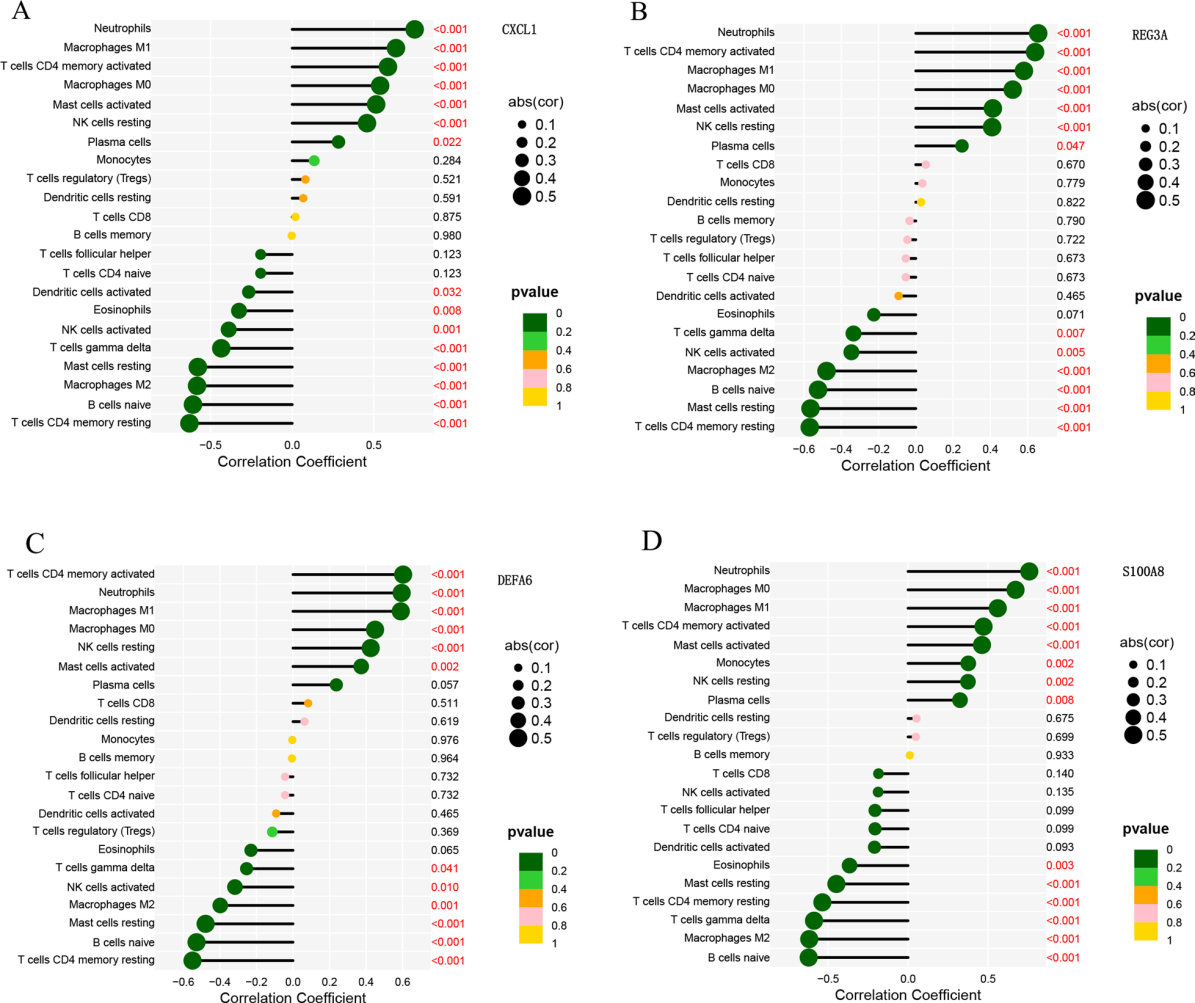
Biomarkers in CD colon tissue. **A**–**D** Correlations among invading immune cells and CXCL1, REG3A, DEFA6, and S100A8. Immune cell names are represented by the vertical coordinates, while correlation coefficients are shown by the horizontal coordinates. The dots' colors and areas correspond to the correlation test's p value and the correlation coefficient's absolute magnitude, respectively. Red is displayed if the *p*-values are less than 0.05

In colon tissue, CXCL1 had a strong positive connection with *neutrophils* (*r* = 0.75, *p* < 0.001) and a significant negative correlation with *T cells CD4 memory resting* (*r* = − 0.62, *p* < 0.001) (Fig. 11A). Neutrophils and REG3A exhibited a positive connection (*r* = 0.65, *p* < 0.001) and a negative correlation with T cells CD4 memory resting (*r* = − 0.56, *p* < 0.001), respectively (Fig. 11B). DEAFA6 positively relationship with *T cells CD4 memory activated* (*r* = 0.60, *p* < 0.001) and negatively relationship with *T cells CD4 memory resting* (*r* = − 0.54, *p* < 0.001) (Fig. 11C). S100A8 was positively connected with *neutrophils* (*r* = 0.75, *p* < 0.001) (Fig. 11D).

**Fig. 12:**
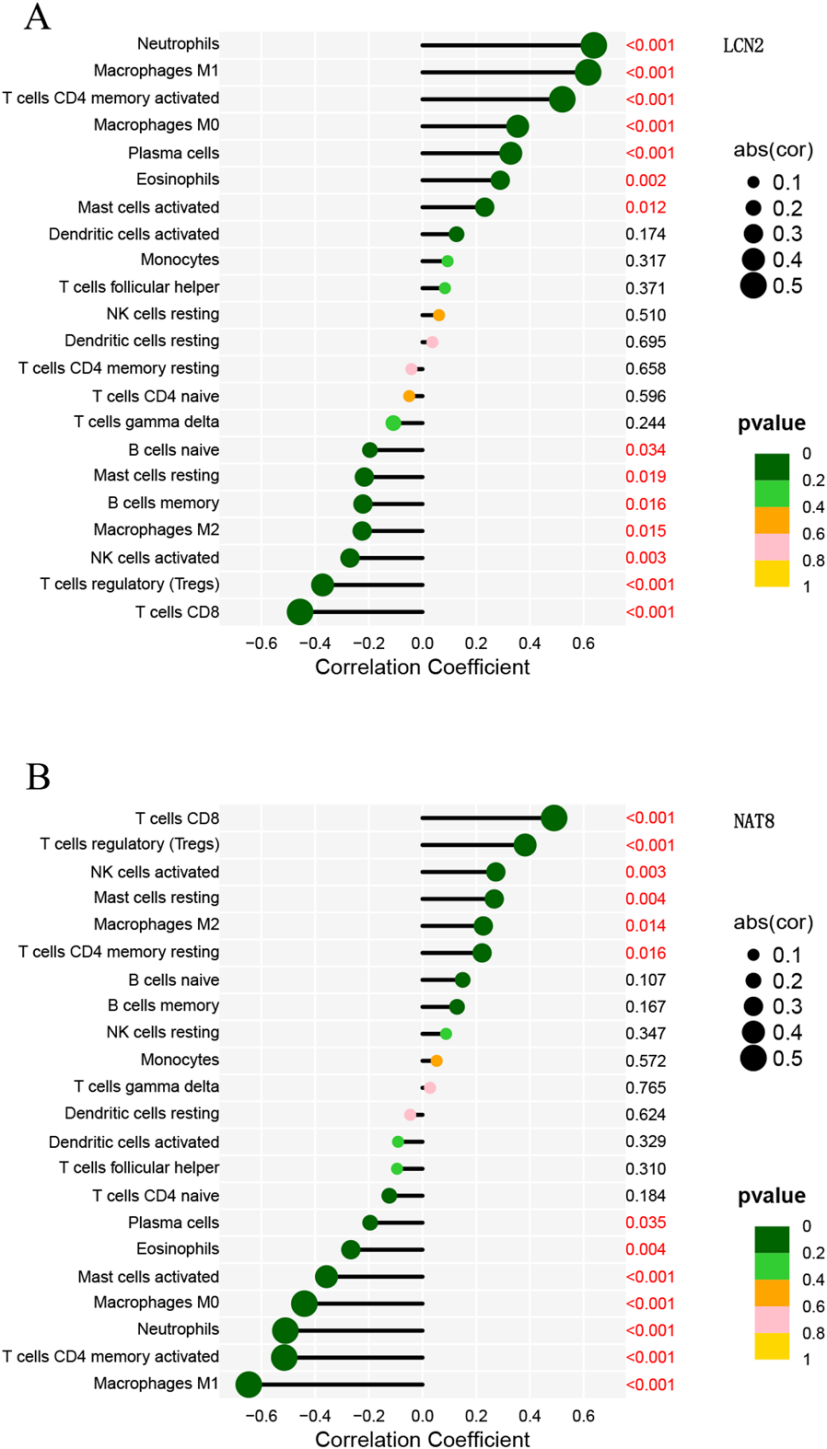
CD ileal tissue biomarkers. LCN2 (**A)** and NAT8 (**B)** correlation with invading immune cells. Immune cell names are represented by the vertical coordinates, while correlation coefficients are shown by the horizontal coordinates. The dots' colors and areas correspond to the correlation test's *p* value and the correlation coefficient's absolute magnitude, respectively. Red is displayed if the *p*-values are less than 0.05

In ileal tissue, LCN2 was positively connected with *neutrophils* (*r* = 0.63, *p* < 0.001) and negatively connected with *T cells CD4 memory resting* (*r* = − 0.45, *p* < 0.001) (Fig. 12A). NAT8 was positively connected with *T cells CD8* (*r* = 0.49, *p* < 0.001) and negatively connected with *macrophages M1* (*r* = − 0.64, *p* < 0.001) (Fig. 12B).

## Discussion

The gradual onset of Crohn's disease (CD), combined with its diverse and non-specific symptoms, can lead to misdiagnosis as other diseases, making it challenging to diagnose accurately. Treatment options are also limited, further complicating diagnosis and treatment and necessitating further research. Additionally, the global incidence of CD is on the rise, and the disease can lead to recurrent progression and disability [27], causing significant patient suffering and long-term healthcare expenses [28]. The development of the disease has been shown to be genetically linked, and researchers are progressively identifying genes associated with inflammatory bowel disease (IBD) [29]. Currently, the primary treatment for CD focuses on relieving inflammation [30]. Rapid advancements in biological information play a crucial role in exploring its pathogenesis, identifying related markers, facilitating pre-disease diagnosis to slow down or reverse intestinal damage, guiding the development of targeted drugs [31, 32], and offering personalized treatment for patients [33].

### Correlation between DEGs and CD

The differentially expressed genes (DEGs) in CD colon and ileal tissues were analyzed using the GEO database's multi-chip association. In total, 34 DEGs were examined in colonic tissues, with 17 showing substantial up-regulation and 17 exhibiting significant down-regulation. A total of 34 DEGs were evaluated in ileal tissues, of which 26 showed considerably higher expression levels, while 8 showed considerably lower levels. According to the available literature, CD is significantly associated with differential genes, including S100A8, NOS2, DUOX2, DUOXA2, APOA1, CEACAM7, CXCL1/9, LCN2, MMP3, MUC1, G6PC, APOB, APOC3, and NAT8. Among them, the S100A8 gene encoding calprotectin is a well-established biomarker for monitoring IBD activity and relapse prediction. However, some limitations remain, such as the lack of guidelines and data on the optimal value of fecal calprotectin [34]. NOD2, a member of the NLR family, is a known risk gene for CD, with NOD2 loss-of-function being closely linked to the disease. Variants of NOD2 have been found to inhibit transcription of the anti-inflammatory cytokine IL-10, and DUOX2 interacts with NOD2 to produce a response in intestinal epithelial cells to bacterial products [35–37]. DUOX2 is a crucial host factor for maintaining intestinal stability, but harmful mutations in this gene may appear before the clinical manifestations of IBD [33]. Studies have shown strong associations between DUOX2 and APOA1 genes and intestinal inflammation [37–39]. In the current study, DUOX2 expression was up-regulated in both ileum and colon of CD patients, which is consistent with the findings of Haberman et al. [40]. Their study demonstrated that DUOX2 and APOA1 are associated with intestinal flora abundance and regulate enterocyte and innate and adaptive immune functions [40]. DUOXA2, a resident endoplasmic reticulum protein, plays a crucial role in DUOX2's maturation and transport from the endoplasmic reticulum [41–43]. In addition, DUOX2, DUOXA2, NOS2, APOA1, CEACAM7, CXCL1, LCN2, MMP3, MUC1, S100A8, G6PC were included in the DUOX2 gene co-expression signature (0.98 <|*r*|< 1); APOB,APOC3,NAT8, CXCL9 were included in the CD-specific APOA1 gene co-expression signature (0.98 <|*r*|< 1), all associated with CD. Furthermore, variants in TNFRSF6B were found to contribute to the pathogenesis of some CD patients, and intervention may be beneficial [35]. CXCL1 exhibits mild increases in non-inflammatory CD mucosa, but high expression in inflammatory CD mucosa [44].

### Analysis of functional correlation

DO enrichment analysis of DGEs in CD colon and ileum tissues primarily focused on intestinal diseases, oral diseases, inflammatory bowel diseases, lung diseases, and immune-related diseases. This is consistent with previous research [6]. CD is frequently coupled with the onset of immune disorders that fall into two primary categories: those triggered by inflammation of the intestinal tract, such as uveitis and iritis [45]. Patients with a family history of IBD may have a higher risk of ocular inflammation due to the shared immune mechanism between the eye and the gut [46, 47]. A second category of autoimmune diseases arises from an increased autoimmune susceptibility, such as primary biliary cirrhosis [48]. Moreover, regarding the extra-intestinal symptoms of CD, lung manifestations are relatively rare. Nevertheless, granulomatous lung disease has been increasingly associated with CD in recent years [49]. Additionally, oral diseases have been reported in CD patients with the prevalence of stomatitis, periodontitis, and oral lesions ranging approximately from 5 to 50% [50, 51].

Analysis of GSEA results revealed that immune response crucially impacts CD, with the enrichments in both colonic and ileal tissues being mainly related to immunity and inflammation. The primary pathways of GSEA enrichment in colon tissue are *Antigen processing and presentation, natural killer cell-mediated cytotoxicity, and cytokine–receptor interaction*. In the ileum tissue, enrichment is *activation of the immune response, adaptive immune response, immune response base on somatic recombination of immune receptors built, cellular response to biological stimuli, and cellular response to molecule of bacterial origin*. Immunity and inflammation are critical in CD pathogenesis. Innate and adaptive immunity is activated at various CD stages, with antigen processing for presentation and natural killer cells playing a vital role in human immune regulation. However, chronic inflammation in the intestinal injury is sustained by chemokines and cytokines [52]. In the inflamed mucosa, immune cells produce cytokines, and the balance between pro- and anti-inflammatory factors influences mucosal healing and development. Additionally, the interaction between cytokine receptors may further impact the overall balance [53].

### Machine learning-based screening of biopotential biomarkers

CXCL1, S100A8, REG3A, and DEFA6 potential biomarkers were ultimately identified in the colon group, while LCN2 and NAT8 were identified in the ileum group through machine learning. The CXC chemokine family includes CXCL1, which attracts the appropriate immune cells. CXCL1 levels were higher in intestinal mucosal tissues of CD patients than in healthy controls, and CXCL1 levels in the intestinal mucosa of active CD were higher than those in remission [54]. S100A8, a small calcium-binding protein highly expressed in neutrophils, can be triggered by specific inflammatory factors [55]. S100A8 induces cytokine secretion from PBMCs to enhance the inflammatory response [56]. In IBD, S100A8 is released and stimulates leukocyte recruitment and cytokine secretion to regulate the inflammatory response [57]. In autoimmune diseases such as CD, this protein is present at high concentrations. Lipocalin-2 (LCN2) is a potent inhibitory protein [58] that plays a role in fatty acid and iron transport, regulation of inflammation, and metabolic homeostasis [59]. Elevated levels of LCN2 in the serum of patients with active CD can be used as a diagnostic biomarker for the active phase [60]. Additionally, LCN2 appears to be up-regulated in the intestinal mucosa of CD patients, possibly related to the protective effect on the intestinal mucosa through the regulation of iron [61, 62]. LCN2 has great potential as a diagnostic marker for CD. DEFA6 is an antimicrobial peptide highly expressed in the small intestinal Paneth cells. Although antimicrobial peptides do not appear to have bactericidal activity, they have been shown to be essential for preventing pathogen invasion of the intestinal tract in several studies [63, 64]. The decrease of DEFA6 in non-inflammatory jejunal tissue of Crohn's patients may be related to the mucosal barrier disorder in these patients [65]. REG3A is overexpressed in colonic tissues of patients with inflammatory bowel disease, and the detection of REG3A in serum could help distinguish mucosal enteropathy from functional enteropathy [66]. However, the predictive value of this protein for inactive inflammatory bowel disease requires further exploration [67]. NAT8 encodes a specific acetyltransferase that is specific to the liver and kidney. Accumulation of NAT8 reduces the level of reactive oxygen species and has an inhibitory effect on colonic adenocarcinoma [12, 68]. The pathophysiology of NAT8 in relation to CD is still unknown and requires more research.

### Immune cell infiltration type

In this study, the deconvolution algorithm CIBERSORT was used to analyze samples from patients with CD and normal samples, revealing a variety of immune cells closely related to CD's biological processes. In colon tissue, *infiltration of neutrophils, macrophages M1, macrophages M0,* and *resting NK cells* increased, while infiltration of resting T cells CD4 memory and naive B cells decreased. In ileal tissues, *infiltration of resting T cells CD4 memory* increased, and infiltration of *naive T cells CD4* decreased. CXCL1, S100A8, REG3A, and DEFA6 were all associated with *neutrophils* in colon tissues after examining the interactions between the selected biomarkers and infiltrating immune cells. In the ileum, LCN2 and NAT8 were associated with *regulatory T cells* (Tregs). These findings highlight the important role played by immune dysregulation in the pathogenesis of CD.

In this study, we utilized a large dataset from the GEO database, comprising colon and ileal tissue samples, to investigate the identification of CD-related diagnostic genes in association with immune cells. We identified a total of six diagnostic gene markers that possess some predictive value for diagnosis. However, the study has certain limitations. First, the retrospective nature of the study precluded the acquisition of timely clinical information. For example detailing whether patients were treated at the time of inclusion in the dataset, also failed to further identify biomarkers for specific disease phenotypes (fibrosis, fistulization). Second, our study included inflammatory tissue samples from colonic and ileal regions; some ileal diagnostic genes were not thoroughly validated due to the limited data available in the GEO database. Therefore, further prospective investigations are needed to determine biomarkers using bioinformatics and to elucidate the role of immune cell infiltration in CD.

## Conclusion

Newly identified putative molecular markers for CD include CXCL1, S100A8, REG3A, DEFA6 (colon), and LCN2, NAT8 (ileum). Neutrophils and CD4 memory resting T cells may play a significant role in CD pathogenesis. Future therapeutic and preventive strategies for CD may increasingly focus on targeting specific immune cells as novel therapeutic approaches.

## Data Availability

Data were deposited into the Gene Expression Omnibus database under accession number GSE20881, GSE75214, GSE179285 and are available at the following URL: https://www.ncbi.nlm.nih.gov/gds.

## Abbreviations

CD: Crohn's disease
DEG: Difference genes
IBD: Inflammatory bowel disease
LCN2: Lipocalin-2
NKG2D: Natural killer group 2 member D
LASSO: Least absolute shrinkage and selection operator
SVM-RFE: Support vector machine-recursive feature elimination
AUC: Area under curve
DO: Gene ontology disease enrichment
GSEA: Gene set enrichment analysis
